# Effective and persistent antitumor activity of HER2-directed CAR-T cells against gastric cancer cells *in vitro* and xenotransplanted tumors *in vivo*

**DOI:** 10.1007/s13238-017-0384-8

**Published:** 2017-03-10

**Authors:** Yanjing Song, Chuan Tong, Yao Wang, Yunhe Gao, Hanren Dai, Yelei Guo, Xudong Zhao, Yi Wang, Zizheng Wang, Weidong Han, Lin Chen

**Affiliations:** 1 Department of General Surgery, Chinese PLA General Hospital, Beijing, 100853 China; 2 Medical School of Chinese PLA, Beijing, 100853 China; 3 Bio-therapeutic Department, Chinese PLA General Hospital, Beijing, 100853 China; 4 Molecular & Immunology Department, Chinese PLA General Hospital, Beijing, 100853 China

**Keywords:** chimeric antigen receptor, HER2, gastric cancer, cancer stem cell, CD137, immunotherapy

## Abstract

Human epidermal growth factor receptor 2 (HER2) proteins are overexpressed in a high proportion of gastric cancer (GC) cases and affect the maintenance of cancer stem cell (CSC) subpopulations, which are used as targets for the clinical treatment of patients with HER2-positive GC. Despite improvements in survival, numerous HER2-positive patients fail treatment with trastuzumab, highlighting the need for more effective therapies. In this study, we generated a novel type of genetically modified human T cells, expressing a chimeric antigen receptor (CAR), and targeting the GC cell antigen HER2, which harbors the CD137 and CD3ζ moieties. Our findings show that the expanded CAR-T cells, expressing an increased central memory phenotype, were activated by the specific recognition of HER2 antigens in an MHC-independent manner, and effectively killed patient-derived HER2-positive GC cells. In HER2-positive xenograft tumors, CAR-T cells exhibited considerably enhanced tumor inhibition ability, long-term survival, and homing to targets, compared with those of non-transduced T cells. The sphere-forming ability and *in vivo* tumorigenicity of patient-derived gastric cancer stem-like cells, expressing HER2 and the CD44 protein, were also inhibited. Our results support the future development and clinical application of this adoptive immunotherapy in patients with HER2-positive advanced GC.

**Electronic supplementary material:**

The online version of this article (doi:10.1007/s13238-017-0384-8) contains supplementary material, which is available to authorized users.

## INTRODUCTION

Gastric cancer (GC) is one of the most common malignant cancers worldwide, especially in China. GC-associated morbidity and mortality, estimated by the National Central Cancer Registry of China (NCCR), ranks second among cancers, and resulted in approximately 679 thousand deaths and 498 thousand new cases in 2015 (Chen et al., 2016). Despite improvements in GC therapy, many patients fail treatment because of tumor recurrence and metastasis. As the predominant treatment for advanced gastric cancer (AGC), chemotherapy is inefficient and toxic because of its nonspecific antitumor activity. Even among patients treated with first-line chemotherapy, the overall response rate (ORR) is less than 50%, and the median overall survival (mOS) is only 6.6 to 18.4 months (Ter Veer et al., 2016). Thus, we focused on adoptive cellular immunotherapy (ACI), which involves the use of genetically engineered T lymphocyte or NK cell treatments for hematological malignancies (Porter et al., 2011).

Chimeric antigen receptor (CAR) T-cell immunotherapy is an ACI in which the CAR-redirected T cells, expressing engineered receptors specific to a particular antigen, are reintroduced into patients and elicit an effective antitumor immune response. CAR-T cells have been in development for 2 decades, beginning with first-generation chimeric genes composed of single-chain variable fragments (scFvs) of monoclonal antibody (mAb) and FcR γ chains or TCR/CD3 ζ chains (Eshhar et al., 1993). Second- and third-generation, with one or more costimulatory molecules, such as CD28, CD137 (4-1BB), ICOS, and OX40, have since been developed. CD19-targeted CAR-T cells display high antitumor activity in children and adults with B-cell malignancies (Park et al., 2016). Additionally, a series of CARs, targeting different antigens, have been constructed and validated in clinical trials. Although this strategy has been effective and presents advantages, such as high specificity and MHC-independent effects, its potential clinical application in solid tumors is hampered by concerns over its safety and efficacy.

Studies on the use of CAR-T cells in the treatment of GC are lacking, mainly because of the high heterogeneity of GC cells. Positive clinical outcomes, obtained using the targeted agent trastuzumab, suggest that the surface antigen HER2 may serve as a target for CAR-T cell therapy. HER2, also known as CD340 or Neu, is a member of the EGFR family, and is encoded by the *ERBB2* proto-oncogene. *ERBB2* is faintly expressed in the luminal and glandular epithelium under normal conditions and is overexpressed in carcinomas of the breast, ovary, endometrium, lung, pancreas, bladder, and stomach (Thibault et al., 2013). Positive rates of HER2 amplification, and overexpression in GC patients, are distinct and range from 10% to 27%, and 8.2% to 53.4%, respectively; this is likely due to differences in methodologies, ethnic groups, pathological types, and tumor locations among the affected patients (Vakiani, 2015). Additionally, *ERBB2* amplification contributes to the maintenance of stem cell (GCSC) subpopulations of gastric cancer (Jiang et al., 2012); the expression status of *ERBB2* is related to disease progression and poor prognosis (Van Cutsem et al., 2016). In this study, we designed novel, lentivirus-mediated, CAR harboring, anti-HER2 scFv, CD3ζ and CD137 signaling domains and evaluated the antitumor activity of CAR-modified T cells against primary GC cells and GCSCs *in vitro* and *in vivo*. Our results support future clinical trials testing the use of this CAR-modified T-cell immunotherapy in patients with AGC and multi-drug resistance.

## RESULTS

### Identification and characterization of novel HER2-targeted CAR-T cells

**Figure 1:**
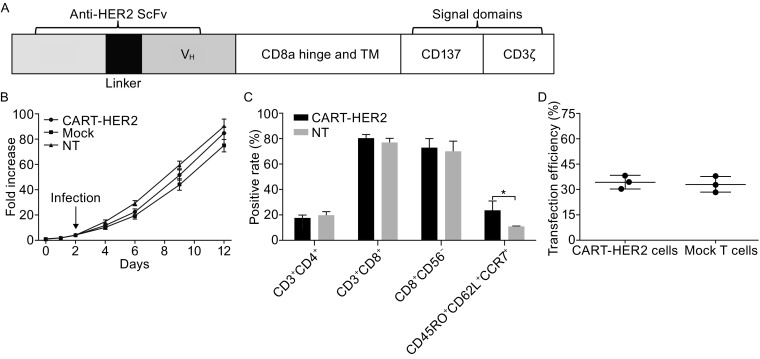
**Generation, expansion, and characterization of HER2-directed chimeric antigen receptor T cells.** (A) Schematic representation of anti-HER2 CAR gene sequence (not drawn to scale). (B) Average expansion (-fold) of CART-HER2, mock T, and NT T cells, produced using PBMCs from three healthy donors; the cells were cultured *in vitro* for 10–14 days. Mean and SDs are shown for three different T-cell lines. (C) Phenotypic features of CART-HER2 and NT T cells, from three healthy donors, were evaluated by FACS analysis on day 12 of culture. Mean positive rates ± SD from three different T-cell lines are shown. (D) Transfection efficiency of *CAR* or mock gene into T cells was determined by FACS analysis using the marker *GFP* on day 12. The data are represented as means ± SD. *Represents *P* < 0.05

A second-generation CAR, consisting of an HER2-specific scFv linked to a hinge domain, a transmembrane (TM) domain, and CD137ζ and CD3ζ moieties in sequence (Fig. 1A), was constructed and inserted into a pseudotyped, clinical-grade lentiviral vector system. A green fluorescence protein (GFP) was added to CAR.HER2-CD137ζ to verify transfection efficiency; CAR-CD137ζ-GFP, without an scFv, was constructed to serve as control.

T cells were originally generated from the peripheral blood mononuclear cells (PBMCs) of three healthy donors. Lentivirus-mediated CAR gene transfection was performed on day 2 of PBMC culture *in vitro*. The total numbers of CAR-transduced, mock-transduced, and non-transduced (NT) cells were expanded on an average of 85, 75, and 90 times, respectively, on day 12 of culture (Fig. 1B). Using fluorescence-activated cell sorting (FACS) analysis on day 12, we determined that the CAR group mainly comprised a large population of CD8^+^CD56^−^ cells (72.55% ± 7.26%) and a small fraction of cells expressing the indicated central memory T (Tcm) phenotypes (CD45RO^+^CD62L^+^CCR7^+^, 23.35% ± 7.59%). The Tcm population was significantly larger than the corresponding population in the NT group (Figs. 1C and S1). Mean transfection efficiencies of 34.22% ± 4.00% and 32.95% ± 4.76% were verified in the final CART-HER2 and mock T-cell populations, respectively (Figs. 1D and S2). Groups of cells, exhibiting optimal growth, were selected from one of the three abovementioned donors, to undergo subsequent functional assays.

### CART-HER2 cells specifically kill HER2^+^ GC cell lines and primary GC cells *ex vivo*

**Figure 2:**
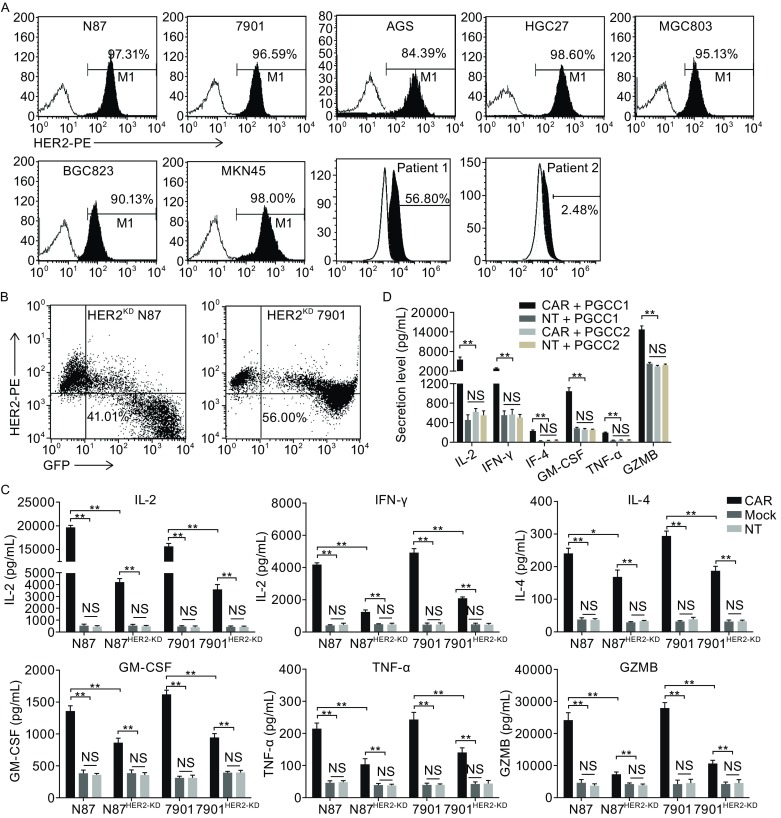
**Specific activity of HER2-directed chimeric antigen receptor T cells against HER2** ^**+**^ **GC cells.** (A) FACS was used to test the surface expression of HER2 proteins in a series of human GC cell lines, including N87, 7901, AGS, HGC27, MGC803, BGC823, MKN45, and primary GC cells from two patients with GC. (B) HER2 expression in N87 and 7901 cells was downregulated via transduction of lentivirus-mediated short hairpin RNA-HER2. The knockdown effects of HER2 expression in sorted GFP-positive cells were evaluated by FACS analysis. (C) The levels of cytokines, released by CART-HER2, mock T, and NT T cells, were measured by enzyme-linked immunosorbent assay (ELISA) after 4-h incubation with HER2^high+^ and HER2^KD^ GC cells at an effector-to-target (E/T) ratio of 20:1. (D) The levels of cytokines, released by CART-HER2 and NT T cells, were measured by ELISA after 4-h incubation with patient-derived GC cells at an E/T ratio of 20:1. The data are represented as the mean cytokine concentrations ± SD (pg/mL) from triplicate cultures. NS represents no statistical significance, *represents *P* < 0.05, **represents *P* < 0.01

FACS was used to assess the surface expression of HER2 proteins in a series of human GC cell lines (GCCLs), including the N87, 7901, AGS, HGC27, MGC803, BGC823, and MKN45 cells, and in primary gastric cancer cells (PGCCs) obtained from two patients with GC. HER2 was strongly expressed in all the GCCLs, with percentages ranging from 84.39% to 98.60%. In contrast, the PGCCs from patient 1 (PGCC 1) exhibited a percentage of 56.80%, and the PGCCs from patient 2 (PGCC 2) exhibited a percentage of 2.48% (Fig. 2A). Therefore, two different GCCLs (N87 and 7901), and two patient-derived cell lines, were selected for further study. HER2 knockdown (HER2^KD^) was performed in N87 and 7901 cells via transduction of a lentivirus-mediated short hairpin RNA-HER2 (shRNA-HER2) (Fig. 2B). Transduction of the *GFP* gene was performed for isolation and tracking. HER2^KD^ tumor cells served as control cells.

To verify the specific activity of CART-HER2 cells against HER2^+^ tumor cells, we incubated T cells with tumor cells at an effector-to-target (E/T) ratio of 20:1 for 4 h. After the incubation, the levels of cytokines released by CART-HER2 cells, including IL-2, IFN-γ, IL-4, GM-CSF, TNF-α, and Granzyme B (GZMB), were significantly elevated in the supernatants of HER2^high+^ N87 and 7901 cells compared with those of mock T cells and NT T cells (Fig. 2C). However, substantially lower cytokine levels were detected when CART-HER2 cells were co-cultured with HER2^KD^ N87 and 7901 cells than when CART-HER2 cells were co-cultured with HER2^high+^ N87 and 7901 cells (Fig. 2C). Similarly, the specific activity of CART-HER2 cells against patient-derived GC cells was verified by incubating the T cells with PGCC 1. Furthermore, minimal levels of cytokines were detected when effector T cells interacted with HER2^−^ PGCC 2 (Fig. 2D). These results indicate that CART-HER2 cells can specifically recognize GC cells with high and low expression of HER2, and can then be activated at varying levels by GC cells.

**Figure 3:**
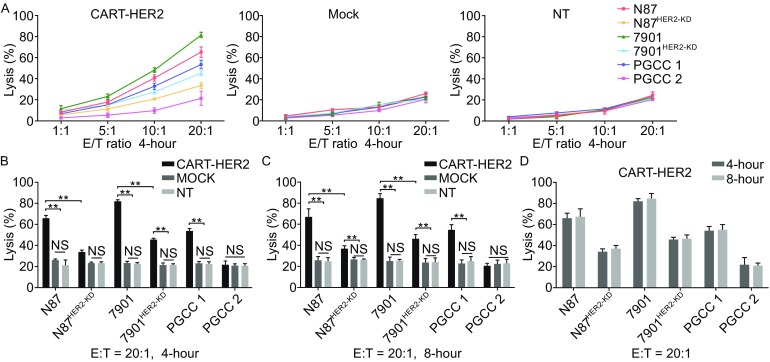
**Specific cytotoxicity exhibited by HER2-directed chimeric antigen receptor T cells against HER2** ^**+**^ **GC cell line and primary GC cells** ***ex vivo***. (A) The cytotoxic activity of CART-HER2, mock T, and NT T cells against HER2^high+^ and HER2^KD^ GC cells was determined using a 4-h lactate dehydrogenase (LDH) release assay in a dose-dependent manner. HER2^−^ primary GC cells were used as controls. (B) A 4-h LDH release assay, at an effector-to-target (E/T) ratio of 20:1, was used to compare cytotoxicity between CART-HER2, mock T, and NT T cells against different GC cells (C) An 8-h LDH release assay, at an E/T ratio of 20:1, was used to compare cytotoxicity between CART-HER2, mock T, and NT T cells against different GC cells. (D) Cytotoxicity comparisons of CART-HER2 cells against different GC cells were performed between the 4-h and 8-h LDH release assays at an E/T ratio of 20:1. All of the results are expressed as the mean of triplicate values ± SD. NS represents no statistical significance, **represents *P* < 0.01

Next, to evaluate the cytotoxicity of CART-HER2 cells against GC cells, we performed time- and dose-dependent lactate dehydrogenase (LDH) release assays. As shown in Fig. 3A and 3B, CART-HER2 cells, evaluated using the 4-h LDH assay, and incubated at a ratio of 20:1 E/T, exhibited higher average killing activity against HER2^high+^ N87 cells (65.3% ± 5.0%), 7901 cells (81.5% ± 2.5%), and PGCC 1 (53.6% ± 4.2%) than did mock and NT T cells having a low rate of lysis. Moreover, the cytotoxicity of CART-HER2 cells was increased as the E/T ratio increased (Fig. 3A). Similarly, the 8-h LDH assay showed that the average killing activity, exhibited by CART-HER2 cells against HER2^+^ GC cells when incubated at a ratio of 20:1 E/T, was higher than that exhibited by mock or NT T cells (Fig. 3C), but was not significantly higher than that exhibited by CART-HER2 cells in the 4-h assay (Fig. 3D). In the control group of target cells, the average killing activity levels, exhibited by CART-HER2 cells against HER2^KD^ N87 and 7901 cells during the 4-h LDH assay when incubated at a ratio of 20:1 E/T, were 33.6% ± 3.0% and 45.3% ± 2.2%, respectively; these levels were still significantly higher than those exhibited by mock or NT T cells (Fig. 3B). There was no significant difference among the three types of effector T cells with respect to cytotoxicity against HER2^−^ PGCC 2 (Fig. 3A). Hence, we demonstrated that CART-HER2 cells possess potent cytotoxic activity against HER2^+^ GC cells.

### CART-HER2 cells exhibit effective and persistent antitumor activity against xenografts derived from HER2^+^ GCCLs in mice

**Figure 4:**
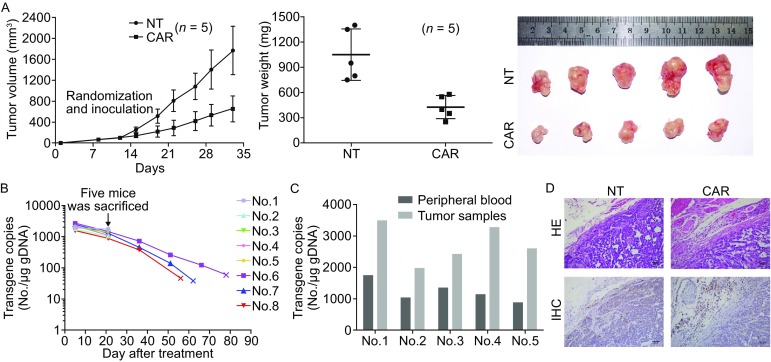
**Antitumor efficacy of HER2-directed chimeric antigen receptor T cells against xenografts derived from HER2** ^**+**^ **GC cell lines in mice.** (A) Two million HER2^high+^ 7901 cells were injected subcutaneously into the unilateral axillary region of BALB/c nude mice on day 0. Five HER2^high+^ tumor-bearing mice per group were randomly assigned to the CART-HER2 and NT groups before treatment. When the mean tumor volume reached approximately 100 mm^3^ on day 12, intravenous treatment with T cells was performed. Mice with maximum tumor diameters over 2 cm were sacrificed on day 33. The tumor volume data are represented as mean ± SD (mm^3^). The tumor weight data are represented as mean ± SD (mg). Images of tumor samples from sacrificed mice are shown. (B) The persistence of the infused CART-HER2 cells in the peripheral blood. qPCR was performed at serial time points, after infusion with CART-HER2 cells, to determine the expression levels of *CAR* in the peripheral blood of mice (*n* = 8). (C) Correlations of *CAR* copy numbers in tumor tissue and blood samples, obtained after the HER2^high+^ mice, treated with CART-HER2 cells, were sacrificed on day 33. (D) Hematoxylin-eosin (HE) and immunohistochemical (IHC) staining for anti-CD3 were performed on tumor samples from sacrificed mice

The subcutaneous xenotransplanted tumor model of HER2^high+^ 7901 cells was established in BALB/c nude mice to determine the antitumor ability of CART-HER2 cells *in vivo*. Treatment with effector cells was performed on day 12, when the mean tumor volume (TV) had reached approximately 100 mm^3^ after subaxillary inoculation with tumor cells. Tumor growth was measured twice weekly until the maximum tumor diameter was greater than 2 cm or until 20% of body weight was lost; the survival time of mice was over 90 days. As shown in Figs. 4A and S3, in the HER2^high+^ mouse model, the rates of tumor growth were considerably inhibited by treatment with CART-HER2 cells, while tumors in the control group continued to grow rapidly after the injection with NT T cells. The mean TV and mean tumor weight (TW) in the CART-HER2 group were 654 ± 247 mm^3^ (*P* < 0.01) and 426 ± 138 mg (*P* < 0.01), respectively, while those in the NT group reached 1,769 ± 462 mm^3^ and 1,050 ± 306 mg, respectively, on day 32. Finally, the significantly improved survival (77 ± 11 days), exhibited by the remaining HER2^high+^ tumor-bearing mice (*n* = 3) treated with CART-HER2 cells, indicated the specific antitumor activity of CART-HER2 cells.

To determine the persistence of the CART-HER2 cells, we used qPCR at serial time points to measure the copy numbers of *CAR* in the peripheral blood of mice in the experimental group. The copy numbers of *CAR* remained at a detectable level for at least 56 days in the blood of the remaining three mice and were positively correlated with the improved survival of HER2^high+^ mice (Fig. 4B).

To assess the homing ability of CART-HER2 cells, transgene copy number detection, as well as hematoxylin-eosin (HE) staining, and immunohistochemical (IHC) labeling, were performed on tumor samples from sacrificed mice. The results showed high levels of DNA copy numbers (Fig. 4C) and considerable increases in the recruitment of human CD3^+^ T cells in the experimental group, whereas only a small number of CD3^+^ T cells were observed in the NT group (Fig. 4D). These findings strongly suggest that CART-HER2 cells can effectively traffic to target sites.

### CART-HER2 cells effectively respond to GCSCs

**Figure 5:**
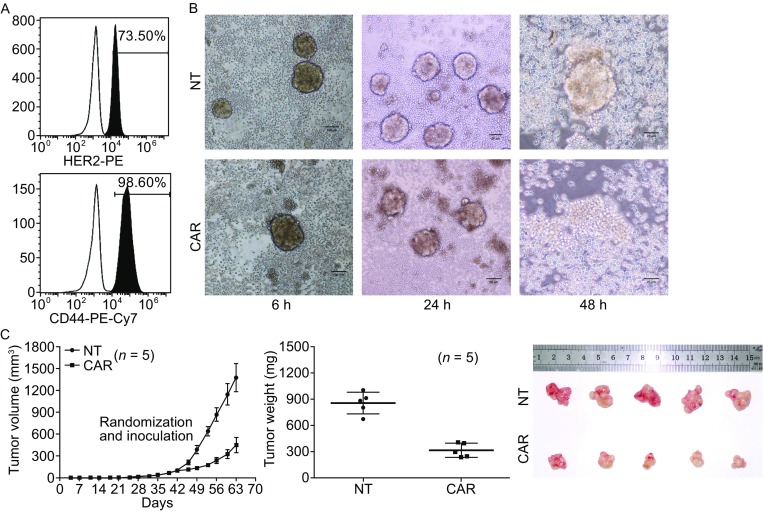
**Effective response by CART-HER2 cells to GCSCs.** (A) FACS analysis was used to determine the surface expression of HER2 and CD44 proteins in gastric cancer stem cells from suspended tumor spheres. (B) Co-culture of tumor spheres with 2 × 10^5^ CART-HER2 cells, or NT T cells, per well was performed in an ultra-low adherent 24-well plate using serum-free media. Images of the killing activity, exerted by CART-HER2 cells against tumor spheres at serial time points, are shown. (C) Ten thousand gastric cancer stem cells were injected subcutaneously into the unilateral axillary region of BALB/c nude mice on day 0. Five mice per group were randomized to the CART-HER2 and NT groups. When the mean tumor volume reached approximately 100 mm^3^ on day 42, intravenous treatment with T cells was performed. Mice with maximum tumor diameters over 2 cm were sacrificed on day 63. The tumor volume data are represented as mean ± SD (mm^3^). The tumor weight data are represented as mean ± SD (mg). Images of tumor samples from sacrificed mice are shown

To verify whether CART-HER2 cells can inhibit the growth of CSC subpopulations in primary GC, suspended cell spheres, which are aggregations of CSCs, were generated in serum-free media containing growth factors. The mean number of spheres per well, in an ultra-low adherent 6-well plate, was 32 on day 20 after inoculation with HER2^+^ PGCCs from patient 1. The rates of the surface expression of HER2, and the GCSCs-specific marker CD44, in separated spheres were 73.5% and 98.6%, respectively, as determined by FACS analysis (Fig. 5A). Then, a co-culture of tumor spheres with 2 × 10^5^ CART-HER2 cells or NT T cells per well, was grown in an ultra-low adherent 24-well plate using serum-free media. CART-HER2 cells migrated to the tumor spheres by chemotaxis after 6 h of co-culture, resulting in the phagocytosis and degradation of the tumor spheres by the CART-HER2 cells. The numbers of spheres in five wells fell by an average of 50% after 24 h. Most of the spheres in the CART-HER group were disintegrated into small pieces after 48 h, indicating that the CART-HER2 cells were significantly more effective than the NT T cells at targeting the tumor spheres (Fig. 5B). The tumorigenicity of the CSCs in the BALB/c nude mice was also remarkably restrained by intravenous treatment with CART-HER2 cells. The average TV and TW in the CART-HER2 group were 450 ± 106 mm^3^ and 310 ± 81 mg, respectively, while those in the NT group were 1,377 ± 196 mm^3^ and 852 ± 124 mg, respectively, on day 63 (Figs. 5C and S4).

## DISCUSSION

To the best of our knowledge, this study is the first to confirm that CART-HER2 cells show specific, effective, and persistent tumor-killing activity against HER2-positive primary GC cells and GCSCs in pre-clinical tests. We designed an anti-HER2 scFv and CD137-containing CAR, which was transduced into T cells by a clinical-grade lentiviral vector system. Our results show that the cytotoxicity, exhibited by CART-HER2 cells against HER2-positive GC cells and HER2-knockdown cells, was significantly higher than that exhibited by NT T cells; this rules out the influence of the vector system on our results and suggests that CAR-T cells contacted the target cells with high affinity and lysed them in an MHC-independent manner. The secretion levels of multiple cytokines were positively correlated with the antitumor effects of CAR-T cells. The cytokines that were upregulated included the pro-inflammatory factors IFN-γ, IL-2, IL-4, GM-CSF, and TNF-α, which are crucial for T-cell activation, proliferation, and differentiation. These cytokines are also implicated in the occurrence of severe cytokine release syndrome (CRS) (Teachey et al., 2016). HER2-positive tumor growth in mice was markedly inhibited by intravenous infusion of CAR-T cells. We detected persistently high copy numbers of *CAR* in the blood and high numbers of infiltrating CD3^+^ cells in tumors, all of which indicated the persistence, homing, and antitumor activity of CAR-T cells. The sphere-forming ability, and *in vivo* tumorigenicity, of primary GCSCs were significantly suppressed by treatment with CAR-T cells; thus, we conclude that CART-HER2 cells can mount an effective response to CSCs.

Components of the HER2 signaling pathway have been used extensively as a key immunotherapy target in therapy against various solid tumors. Although several anti-HER2 drugs, such as trastuzumab, pertuzumab, lapatinib, and TDM-1, have been tested in clinical GC trials, only trastuzumab is currently approved for the first-line treatment of AGC (Fanotto et al., 2016). Hence, to enhance treatment efficacy and minimize drug resistance, the HER2-directed CAR approach has been developed and validated in diverse tumor-bearing mouse models, including those of osteosarcoma (Rainusso et al., 2012), breast cancer (Sun et al., 2014), renal cancer (Schonfeld et al., 2015), and glioblastoma (Zhang et al., 2016). Recently, the first clinical study to use HER2-specific CAR-T cells in patients with HER2-positive sarcoma showed that the HER2-directed CAR approach performed well with respect to safety and efficacy (ORR, 7/17) (Ahmed et al., 2015). These data suggest that HER2 may be a suitable target for our CAR-modified T-cell immunotherapy. In this study, we demonstrated that CART-HER2 cells have high affinity for all types of GC cells, even those expressing low levels of HER2, and display an effective and persistent killing activity in HER2-positive tumor-bearing mice.

Additional costimulatory domains, incorporated into CARs, are indispensable for engineering T cells that show a potent and sustained antitumor activity (Dai et al., 2016). Despite the existence of multiple alternative costimulatory molecules, the majority of modular designs, used in clinical trials, are based on the CD137 and CD28 signaling domains. T cells expressing CD137-containing CARs are likely to show higher proliferation and persistence than those expressing CD28-containing CARs (Zhang et al., 2015). The mechanism underlying these functional differences remains unclear. One study showed that the early exhaustion of T cells, induced at varying levels by an antigen-independent tonic signaling caused by CAR clustering, was ameliorated by CD137 domains. This may have occurred via inhibition of the expression of exhaustion-related genes and overexpression of the genes regulating metabolism, apoptosis, and responsiveness to hypoxia (Long et al., 2015). Similarly, another study showed the pivotal role of CD137 signaling-induced metabolic reprogramming in the enhanced persistence of CAR-T cells. These metabolic changes, including the considerable enhancements in respiratory capacity, fatty acid oxidation, and mitochondrial biogenesis, result in the differentiation of central memory and prolonged survival of CAR-T cells (Kawalekar et al., 2016). The results of these studies agree with our findings of increased proportions of CD45RO^+^CD62L^+^CCR7^+^ cells, and long-term survival of CAR-T cells, *in vivo*. However, CD28 CARs exert greater cytotoxic effects on T cells than do CD137 CARs. Combining these two domains, or their ligands, results in higher antitumor efficacy in some mouse models (Zhao et al., 2015); however, this structural design warrants further clinical study.

Transduction of *CAR* into primary T cells is accomplished using a series of gene transfer platforms, ranging from a plasmid vector- to virus-mediated gene delivery; each approach offers its pros and cons with respective efficiency, toxicity, practicability, and cost. The recombinant plasmid was initially used for the genetic modification of T cells because of safety concerns; although this method is associated with a low risk of transgene-specific immune responses and insertional mutagenesis, it delivers low transfection efficiency and limited cell survival (Jensen et al., 2000). Another approach, not involving transgene integration, is the mRNA electroporation system, which exhibits very high transfection efficiency and facilitates rapid, but transient, *CAR* expression in T cells (Zhao et al., 2006). Recently, the Sleeping Beauty (SB) and PiggyBac (PB) transposon/transposase systems, which can integrate genes into target cellular genomes, were evaluated in CAR-related therapies. The low efficiency of genetic transposition is a major obstacle preventing the broad clinical application of this method; however, the low cost, convenient manufacturing, and mild toxicity of this system render it attractive as an alternative treatment strategy (Kebriaei et al., 2016). Surprisingly, one study demonstrated a markedly enhanced CAR transduction using a minicircle vector carrying an SB system (Monjezi et al., 2017). Because they are currently the most widely used vectors, retroviral and lentiviral vectors allow for the stable and efficient transfection of *CAR* into T cells. The ability of these modified cells to elicit a well-tolerated, effective, and persistent therapeutic response has been proven in patients with different types of cancer (Jackson et al., 2016).

Tumors possess a hierarchical structure, in which CSCs serve as the tumor-initiating cells and are responsible for drug resistance, tumor progression, recurrence, and metastasis; thus eradication of CSCs is a promising method for treating cancer (Pan et al., 2015). GCSCs have also been identified using specific markers, such as CD44 (Takaishi et al., 2009); the stemness properties of GCSCs are studied using two standard approaches: the *in vitro* sphere-forming assay and *in vivo* serial tumor passaging (Brungs et al., 2016). Some studies have found that HER2 plays a critical role in maintaining the CSC subpopulation of GC cells (Jiang et al., 2012), as well as that of other tumor cells, such as breast tumor cells (Korkaya and Wicha, 2013). Therefore, because of the eradication of CSCs, HER2-targeted therapy may result in improved treatment outcomes in patients with HER2-positive GC. In this study, we used sphere cultures *in vitro*, and tumor formation in mice, to successfully validate the self-renewal and tumor-initiating capacity of CD44^+^HER2^+^ GCSCs from primary gastric tumors. We found that CART-HER2 cells efficiently phagocytized and degraded the tumor spheres and suppressed the growth of xenograft tumors in nude mice inoculated with GCSCs. These results show that CART-HER2 cells exerted potent cytotoxic effects on GCSCs and suggest that CAR-T cells are potentially effective in preventing the recurrence and metastasis of GC. However, the practical impact of this treatment needs to be verified further in future clinical trials, given the genetic and functional heterogeneity of CSCs (Kreso and Dick, 2014) and the complexity of the tumor microenvironment (Plaks et al., 2015).

Although the therapeutic effects of CAR-T cells on hematological malignancies, especially CAR-T cells targeting CD-19, have been elucidated, the development of CARs for the treatment of solid tumors has been hindered by the paucity of optimal antigens, the inability of T cells to migrate to and infiltrate primary lesions, transgene immunogenicity and toxicity, ongoing evolution of cancer cells, possible existence of CSC populations, and the immunosuppressive tumor microenvironment. Therefore, additional methods for implementing CAR-T cell therapy against solid tumors have been explored. Modifying T cells with inducible cytokine or chemokine receptor transgenes has been shown to enhance their antitumor activity and ability to localize. For example, supplementary secretion of IL-12 by T cells, engineered with CAR and CAR-inducible *Il-12* genes in targeted sites, improved the intrinsic functions of these T cells and boosted the local immune response by facilitating the recruitment and activation of other killing cells, the initiation of innate immunity, and modulation of suppressor cells (Chmielewski et al., 2014). Similarly, synchronous blockade of PD-1 in CAR-T cells, via genetic modification, also prolonged cell survival (Cherkassky et al., 2016) and augmented tumor-killing efficacy in mouse tumor models (Liu et al., 2016). Another approach involves designing more intelligent CAR constructions to minimize the off-target effects. These constructions include bispecific CAR-T cells, which can be activated only by the identification of both CAR molecules (Wilkie et al., 2012), and the synNotch circuit, in which T cells are armed only by a synthetic Notch receptor activated by one target to induce the expression of a CAR against another target (Roybal et al., 2016). Additionally, combining CAR-T cells with ibrutinib in patients with leukemia (Fraietta et al., 2016) may represent a potential strategy for enhancing therapeutic efficacy against solid tumors.

In conclusion, we showed that HER2-directed CAR-T cells exhibit effective and persistent antitumor activity against patient-derived GC cells, and GCSCs, *in vitro* and *in vivo*. Our results suggest that these modified T cells may be applicable in patients with HER2-positive GC; however, the toxicity and immunogenicity of CAR need to be addressed in future clinical trials.

## MATERIALS AND METHODS

### Cells

The human GCCLs, N87, 7901, AGS, HGC27, MGC803, BGC823, and MKN45 were obtained from the Institute of Basic Medicine of Chinese PLA General Hospital (PLAGH). PGCCs were obtained from the Department of General Surgery of PLAGH. All GC cells were cultured in RPMI-1640 medium supplemented with 10% fetal bovine serum (FBS), except the MGC803 cells, which were cultured in DMEM (high glucose) containing 10% FBS.

### Isolation of primary gastric tumor cells

Fresh human GC tissues were obtained from patients immediately after resection. All samples were transported on ice to the laboratory within 30 min, and were immediately mechanically disaggregated into 1-mm pieces using scissors. These tumor pieces were then digested with 1 mg/mL collagenase I and 1 mg/mL collagenase IV (Life Technologies, Waltham, MA, USA), and diluted in phosphate buffered saline (PBS) at 37°C for at least 1 h. Digestion was terminated with DMEM/F12 containing 20% FBS (Life Technologies).

### Tumorsphere culture and formation

To form spheres, digested cells from primary gastric tumors were cultured at a density of 10^4^ cells per well in ultra-low attachment 6-well plates (Corning, NY, USA) in DMEM/F12 supplemented with 2% B27 (Invitrogen, CA, USA), EGF (20 ng/mL, Invitrogen), bFGF (10 ng/mL, Invitrogen), LIF (10 ng/mL, Peprotech, Hartford, CT, USA), and HEPES (Invitrogen). The culture medium was replenished twice per week. Spheres with a diameter >100 µm were counted 14 days after being seeded.

### Constructs and lentiviral packaging

The scFv nucleotide sequence, targeting HER2 antigens, was derived from our proprietary product (patent CN105384824A, China). The entire DNA sequence of CAR.HER2-CD137ζ, which contained anti-HER2 scFv, human CD8a hinge, and CD137ζ and CD3ζ signaling domains, was constructed based on our previously published CAR sequence (Dai et al., 2015). Complete clinical-grade viral particles were produced using transfection package cells with a four-plasmid system. The GFP-harboring vector CAR.HER2-CD137ζ-GFP was also constructed to verify transduction efficiency.

### Generation and expansion of CART-HER2 cells

Anticoagulated fresh blood was obtained from three healthy donors. PBMCs in buffy coats were purified using lymphocyte separation medium (Cedarlane), via density gradient centrifugation, after the serum was collected from the blood samples. The washed PBMCs were directly added to the lymphocyte culture medium (Takara) supplemented with the anti-CD3 mAb OKT3 (500 ng/μL) and IL-2 (300 U/mL). The generation and expansion of CAR-T cells was performed in accordance with our published protocol pertaining to cytokine-induced killer (CIK) cells (Wang et al., 2013). Stable transfection of T cells with lentivirus-mediated CAR was conducted on day 2 of cell culturing. Transfection efficiency and phenotypic properties of the CAR-T cells were evaluated by FACS analysis on day 12.

### HER2 knockdown by lentivirus-mediated shRNA

Knockdown of HER2 expression in N87 and 7901 cells was performed by transfection with a lentivirus harboring human HER2-specific shRNA and the *GFP* gene. The lentivirus was purchased from GenePharma. The target sequence for HER2 was 5′-GGAAGGACATCTTCCACAAGA-3′. HER2 knockdown was confirmed by qPCR and FACS analysis.

### Flow cytometry analysis and cell sorting

All of the anti-human antibodies used in this study, including HER2 (phycoerythrin, PE), CD3 (chlorophyll protein complex PerCP), CD4 (fluorescein isothiocyanate, FITC), CD8-PE, CD45RO (allophycocyanin, APC), CD56-APC, CD62L-PE, CCR7-PE-Cy7, and CD44-PE-Cy7, were purchased from BD Biosciences. Isotype-matched control mAbs were applied in all the procedures. Additionally, GFP-positive HER2^KD^ N87 and 7901 cells were separated from untransfected cells, based on the degree of fluorescence, by a BD Influx cell sorter (BD Biosciences). FACS data were analyzed by a FACSCalibur flow cytometer (BD Biosciences) and FlowJo software (Version 10.0.7, FlowJo, Ashland, OR).

### Cytotoxicity assays

The specific *in vitro* antitumor activity of CART-HER2 cells was evaluated using a standard LDH release assay (Promega). Briefly, the different types of target cells were seeded in triplicate 96-well plates, at a density of 10^4^ cells per well, with 50 μL of medium; then, an equal volume of effector cells, or control medium, was added to each well to ensure an E/T ratio of 20:1, 10:1, 5:1, or 1:1. After 4-h and 8-h co-incubation, cell supernatants were obtained by centrifugation. Fifty microliters of sample from each well was transferred to new plates to measure the absorbance signals using a plate reader (Multiskan MK3, Thermo Scientific); the remaining samples were collected for cytokine measurements. The average cytotoxicity percentage was calculated according to the formula provided by Promega.

### Cytokine secretion assays

The harvested samples of supernatant, obtained from the 4-h co-incubation of effector cells with target cells at an E/T ratio of 20:1, were assessed for the levels of cytokine secretion. The concentrations of IL-2, IL-4, TNF-α, IFN-γ, GM-CSF, and GZMB were batch-measured using an enzyme-linked immunosorbent assay kit (BD Biosciences) in accordance with the operating manual.

### Quantitative real-time PCR

To determine the levels of CAR.HER2-CD137ζ gene expression, real-time PCR was performed using an ABI PRISM 7900HT Sequence Detection System (Applied Biosystems) according to a procedure published previously (Till et al., 2012). A 153-base pair (bp) DNA sequence, harboring fragments of the CD8a and CD137ζ moieties, was amplified using the following primers: 5′-GGTCCTTCTCCTGTCACTGGTT-3′ and 5′-TCTTCTTCTTCTGGAAATCGGCAG-3′. The transcription level of *HER2* in HER2^KD^ N87 and 7901 cells was quantified via amplification of an 86-bp fragment using the following primers: 5′- ATATATCGAGGCGATAGGGTTAAGG-3′ and 5′-CCGGGGCATATCTTCTGGAAT-3′. β-Actin was used as a control for normalization.

### Xenograft mouse model of GC

The animal experiments were approved by the Animal Ethics Committee of PLAGH. BALB/c nude mice were purchased from the Beijing Vital River Laboratory Animal Technology Co. Four- to 5-week-old female BALB/c nude mice were bred under specific pathogen-free conditions in the Laboratory Animal Center of PLAGH. One hundred microliters of 2 × 10^6^ HER2^high+^ 7901 cells was injected subcutaneously into the unilateral axillary region of BALB/c nude mice on day 0. The rate of xenotransplanted tumor formation was 100%. At least five HER2^high+^ tumor-bearing mice were randomly assigned to the CART-HER2 and NT groups before treatment. The TV of each mouse was measured twice weekly using a vernier caliper, and was calculated according to the following formula: TV = 1/2 × length × width^2^. On day 12, when the mean TV reached approximately 100 mm^3^, 200 μL of 2 × 10^7^ CART-HER2 cells, or NT T cells, were infused into the caudal veins of the mice. For the detection of *CAR* copy number, peripheral blood samples were collected from the caudal veins, or eyeballs, of HER2^high+^ mice, treated with CART-HER2 cells, every 15 days. Inoculation with GCSCs and treatment in BALB/c nude mice were performed as described above. The total number of injected CSCs was 1 × 10^4^, and the tumor formation rate was 100%. Five mice per group were randomized to the CART-HER2 and NT groups. Mice with maximum tumor diameters over 2 cm, or 20% loss of body weight, were euthanized.

### Histological and immunohistochemical examinations

Tumor tissue samples, which had been resected from sacrificed mice, were fixed in 4% paraformaldehyde for 4–6 h, dehydrated in ethyl alcohol, and embedded in paraffin, before being cut into multiple 6-μm thin sections using a microtome. HE and IHC staining were performed using standard procedures. Primary rabbit anti-human CD3 antibody (ab5690, Abcam) and a secondary biotinylated goat anti-rabbit antibody were used for IHC staining.

### Statistical analysis

All results are reported as the mean ± standard deviation (SD), and were analyzed by the unpaired *t*-test. Data were plotted using GraphPad Prism software version 6.0c for Mac OS. *P*-values < 0.05 were considered statistically significant.

## Electronic supplementary material

Below is the link to the electronic supplementary material.

Supplementary material 1 (PDF 696 kb)
