# Supplementary material for: Effective and persistent antitumor activity of HER2-directed CAR-T cells against gastric cancer cells in vitro and xenotransplanted tumors in vivo
Source: Protein Cell. 2017 Mar 10;9(10):867–78. doi: 10.1007/s13238-017-0384-8 (PMC6160382; doi:10.1007/s13238-017-0384-8)
Supplement: Supplementary file 1 — Supplementary material 1 (PDF 696 kb) [file 13238_2017_384_MOESM1_ESM.pdf]

Supplementary Materials

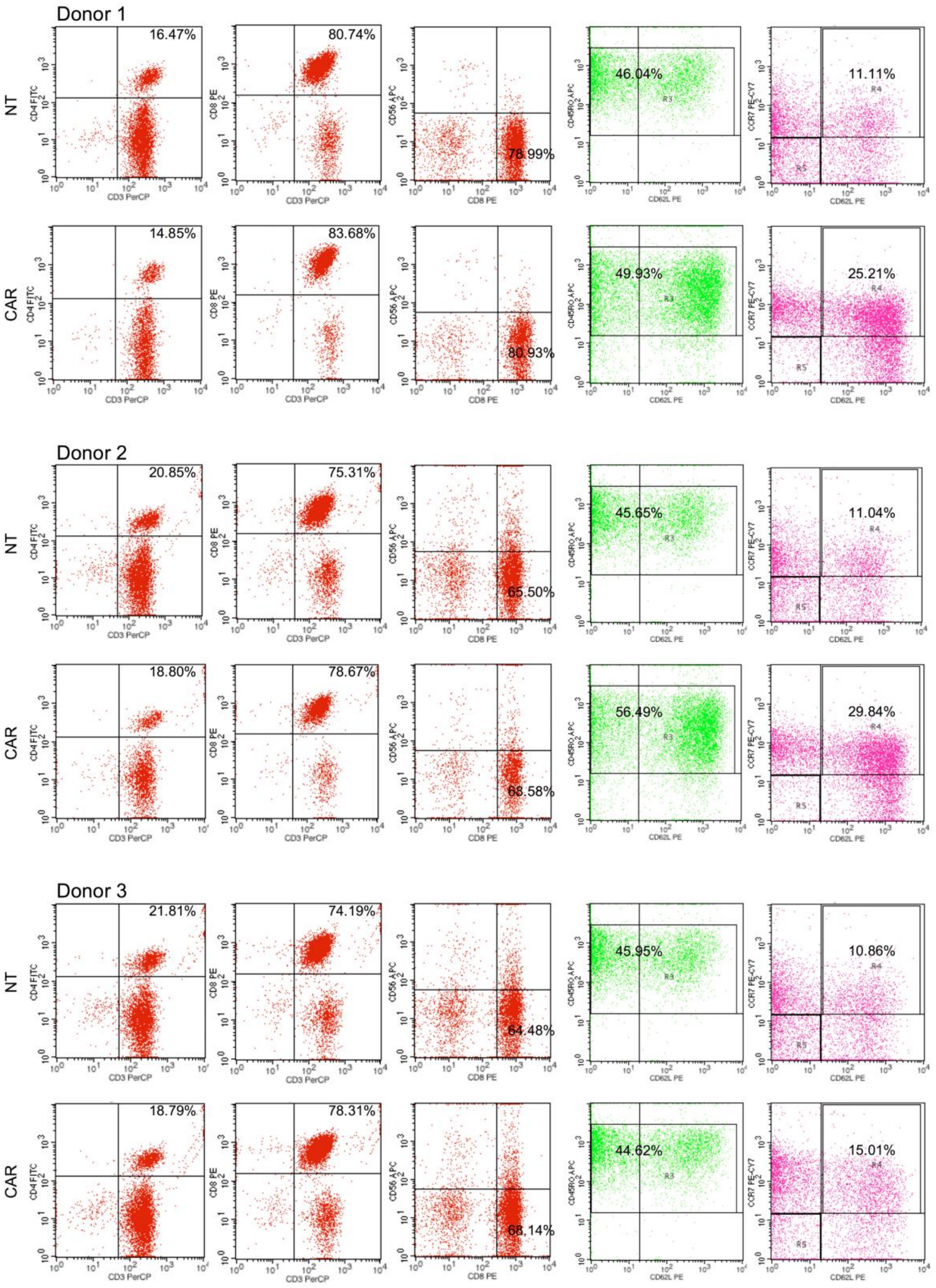

Figure S1. Phenotypic characteristics of CART-HER2 and NT T cells, from three healthy donors, were determined by FACS analysis on day 12 of culture.

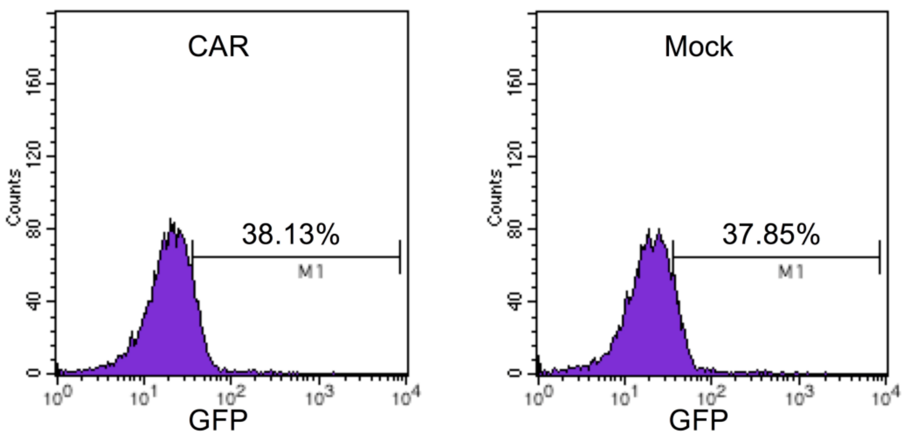

Figure S2. Transfection efficiencies of CART-HER2 and mock T cells, selected from one of the three healthy donors, were measured by FACS analysis using the marker GFP on day 12 of culture.

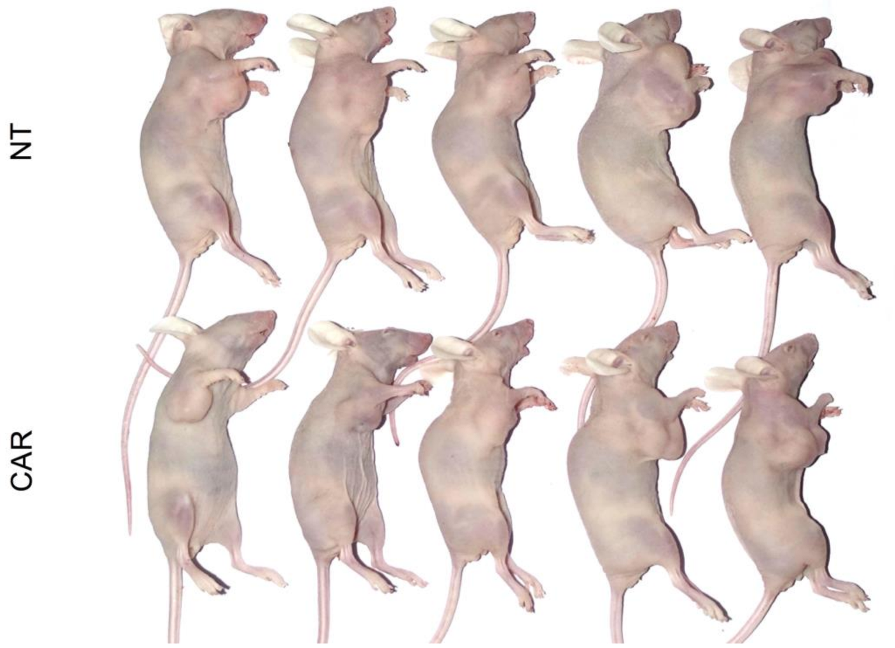

Figure S3. Images of HER2^{high+} tumor-bearing mice treated with CART-HER2 and NT T cells; the mice were sacrificed on day 33 after inoculation with tumor cells.

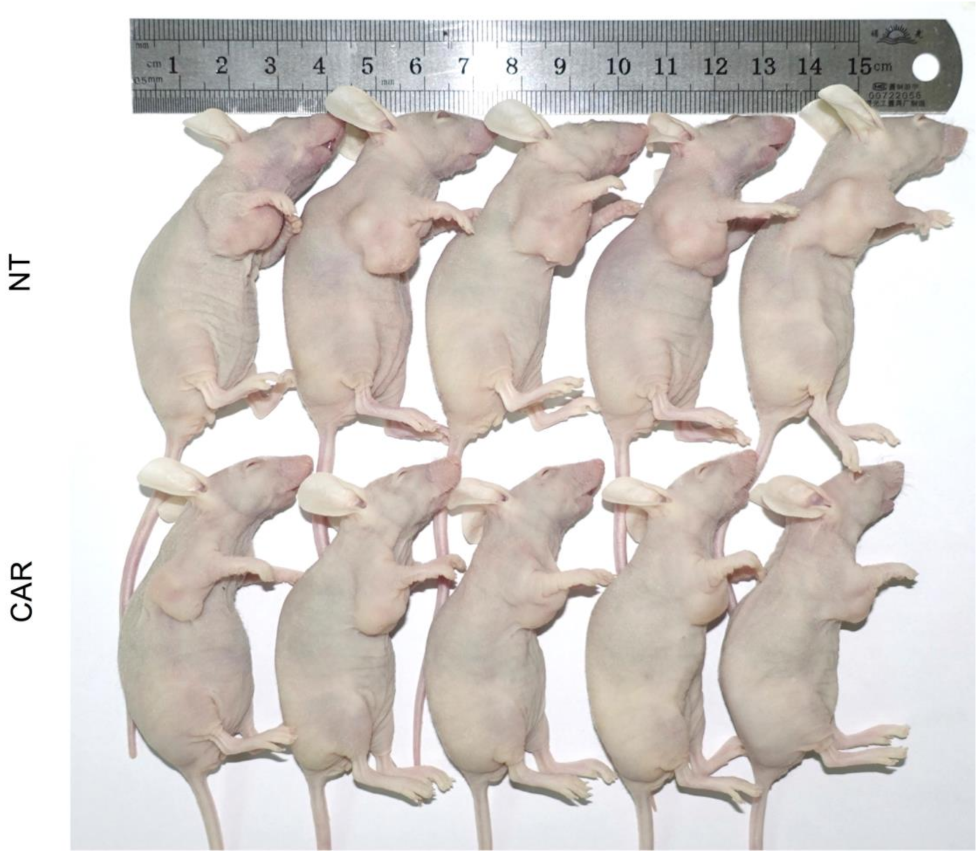

Figure S4. Images of mice with GCSCs-derived tumors, sacrificed on day 21 after treatment with CART-HER2 and NT T cells.
